# Differences in total stored C and N in dryland red soil caused by different long-term fertilization practices

**DOI:** 10.1038/s41598-022-10864-y

**Published:** 2022-04-28

**Authors:** Weifeng Xi, Kailou Liu, Xichu Yu, Xinpeng Xu, Shicheng Zhao, Shaojun Qiu, Ping He, Wei Zhou

**Affiliations:** 1 Key Laboratory of Plant Nutrition and Fertilizers, Ministry of Agriculture and Rural Affairs, Institute of Agricultural Resources and Regional Planning, Chinese Academy of Agricultural Sciences, Beijing, 100081 China; 2 Scientific Observational and Experimental Station of Arable Land Conservation in Jiangxi, Ministry of Agriculture, Jiangxi Institute of Red Soil, Nanchang, 331717 China

**Keywords:** Biogeochemistry, Environmental sciences

## Abstract

Fertilizer application is important to achieve sustainable agriculture. However, it remains unclear about the effects of long term fertilization on C and N immobilization as well as C/N ratios in soil aggregates at different depths. Samples taken at depths of 0 to 40 cm from dryland red soil subjected to long-term fertilization were analyzed. Four treatments were involved in the long term fertilization including no fertilizer (control), chemical fertilizer applied at two different rates, and manure combined with chemical fertilizers (MNPK). The C and N concentrations in the soil aggregates of different sizes were significantly higher (P < 0.05) and the C/N ratios in the particulate organic matter were significantly lower (P < 0.05) for soil to 20 cm deep for the MNPK treatment than for the other treatments. ANOVA indicated that the C and N concentration and C/N ratios in different sizes of aggregates significantly varied with soil depth (P < 0.05). Microaggregates contained most of the C and N, and the C/N ratios for silt–clay particles in macroaggregates were 1.37 unit (ranging − 0.25 to 2.44) lower than for other soil particles with diameters < 53 µm. The C and N contents in aggregates of different sizes increased as the C input rate increased to a depth of 40 cm because of the fertilization practices. Overall, both increased C input and deep application of C sources promoted the storage of C and N in microaggregates, which in turn increased C and N sequestration in dryland red soils.

## Introduction

Soil organic matter (SOM) content, and soil organic carbon (SOC) and soil organic nitrogen (SON) contents in particular, are important indicators of soil fertility and health^1,2^. Global warming as a result of emissions of CO_2_ and reactive N compounds is drawing increasing attention to C and N stocks in soil^3–5^. Physical protection of the organic matter in soil aggregates is widely accepted as being mainly responsible for the long-term maintenance of the SOM stock^6–8^. Optimal fertilizer application practices promote the increase of SOC in aggregate fractions, which helps regulate the mineralization and immobilization of N in soil^9,10^. It is therefore necessary to understand whether the mechanisms involved in the physical protection of C and N in soil subjected to different fertilization practices are consistent.

In intensive agricultural systems, applying both chemical fertilizers and manure is an effective way of improving C and N stocks in soil. Applying manure provides C and promotes immobilization of N by microorganisms, and the available nutrients in manure can substantially replace chemical fertilizers and decrease chemical fertilizer use in agriculture^4,11,12^. In parallel, chemical fertilizers can enhance soil C and N stocks through crop residues returned to soil^4,13^. Overall, soil C and N accumulation occur when the C and N inputs exceed outputs and losses via runoff, leaching, or gases emission. Active/dissolved C and N in soil are readily leached and transferred to the deep soil, where they can be absorbed by deep soil particles or further leached out of the root zone^14–16^. It is therefore important to assess the potential for C and N to be sequestered by agricultural surface and subsurface soil subjected to various long-term fertilization practices.

Aggregates in soil can be divided into various size classes. Macroaggregates (> 250 µm diameter) are crucial to SOC stabilization. Macroaggregates consist of microaggregates (53–250 µm diameter) and silt–clay particles (< 53 µm diameter) bound together by plant and microbe residues^17,18^. Microaggregates within macroaggregates can sequester C for a long time and are bound together by well-decomposed plant and microbe residues. C in microaggregates within macroaggregates is more stable than C in other soil fractions and is protected from mineralization by microbes^10,19^. C is also present in silt–clay particles as organo-mineral associations held together through adsorption to mineral surfaces or cation bridging^20,21^. Plant residues not only bind macroaggregates but are also the main forms of particulate organic matter (POM) in soil; besides, POM provides nuclei for aggregation formation^22,23^. C and N in bulk soil can be divided into a labile pool and a stable pool. POM is an important part of the labile pool and is involved in short-term C and N cycling. C and N stocks vary with soil depth and are higher in surface soil than subsurface soil because more exogenous substrate amendments (e.g., fertilizers and crop residues) are mainly present in surface soil. Generally, smaller amounts of C and N are contained in aggregates of various sizes in subsurface soil than in surface soil, so more sorption sites should be available on mineral surfaces in subsurface soil than surface soil^24^. However, little information is available about how C and N in aggregates of different sizes at different depths respond to active C and N from exogenous manure or fertilizer leaching from the surface soil to subsurface soil under different fertilization practices.

The C/N ratio is an important indicator of soil quality. The C/N ratio of bulk soil is generally constant, and the availability of N in soil is better indicated by the C/N ratios of different soil pools than by the C/N ratio of the bulk soil^25^. The C/N ratios of stable fractions in aggregates of different sizes combine to give a stable C/N ratio for bulk soil, but the C/N ratio of POM reflects the plant residue quality and the degree to which the plant residues have decomposed^4,18,25,26^. The C/N of exogenous sources of C (e.g., straw or manure) determines the C/N ratio of POM, and N immobilization occurs when the C/N ratio is greater than 25^27,28^. However, C and N are protected to different degrees in aggregates of different sizes; an understanding of the C/N ratios of aggregates of different sizes is required to determine which soil aggregate fractions contain the largest amounts of available N.

Red soil (Cambisol) is mainly found in South China. It accounts for 23.3% of all Chinese agricultural soil, and upland red soil accounts for 11.3% of all upland soil in China. Red soil is characterized by low organic matter content, low concentrations of available nutrients, poor structure, low pH, and high aluminum content^29^. The application of manure and chemical fertilizers can improve the SOC and SON concentrations in red soil. However, high temperatures and rainfall in the subtropical and tropical areas in which red soil is found cause SOM to decompose rapidly and dissolved C and N to be leached from the soil. The aim of this study was to improve our understanding of physical protection of C and N in aggregates in soil by (1) investigating changes in the C and N concentrations of aggregates and the C/N ratios in soil to a depth of 40 cm after various long-term fertilization practices and (2) evaluating the potentials for C and N to be stored in different aggregates after various long-term fertilization practices.

## Results

As shown in Table 1, the macroaggregate and fPOM masses in soil 10–20 cm deep and the inter-SC masses at 0–10 and 10–20 cm deep were significantly higher (P < 0.05) for the fertilizer treatments than for the control. However, the free-m masses at 0–10 and 10–20 cm deep and the free-SC mass at 0–10 cm deep were significantly lower (P < 0.05) for the fertilizer treatments than for the control. The macroaggregate and cPOM masses at 0–10 cm deep, fPOM masses at 10–20 cm deep, and fiPOM and inter-SC masses at 0–10 and 10–20 cm deep were significantly higher (P < 0.05) for the MNPK treatment than for the chemical fertilizer treatments. The cPOM and fPOM masses at 0–10 cm deep were significantly higher (P < 0.05) for the 2NPK treatment than for the NPK treatment. At all depths, macroaggregates contributed more than the other fractions to the total mass of all water-stable aggregate fractions. The dominant fraction in macroaggregates was inter-m.

**Table 1:** Masses of water-stable aggregates and macroaggregate fractions to 40 cm deep in the control treatment (CK), chemical fertilizer treatments (NPK and 2NPK), and combined manure and chemical fertilizer treatment (MNPK) (unit = %).

Treatment	Water stable aggregates			Macroaggergates			Density fractionation		
	Macro	Free-m	Free-SC	cPOM	Inter-m	Inter-SC	fPOM	fiPOM	Intra-SC
**0–10 cm**									
CK	50.8b	40.0a	8.0a	0.20c	24.2c	19.5c	0.052b	0.023b	23.4c
NPK	59.2b	34.6b	5.1b	0.26c	28.8ab	24.3b	0.058b	0.061b	28.4ab
2NPK	59.4b	34.0b	4.7b	0.41b	30.0a	23.6b	0.081a	0.068b	29.4a
MNPK	61.0a	32.1b	5.2b	0.80a	25.5bc	28.5a	0.084a	0.219a	24.7bc
**10–20 cm**									
CK	67.5c	27.3a	4.0a	0.18a	40.3a	22.2c	0.025c	0.021b	39.8a
NPK	71.2b	23.6b	3.9a	0.23a	39.7a	26.1b	0.030bc	0.022b	39.2a
2NPK	73.1ab	23.2b	2.9a	0.21a	39.7a	27.1b	0.039b	0.036b	39.3a
MNPK	75.3a	20.2c	3.1a	0.24a	40.2a	30.1a	0.054a	0.095a	39.5a
**20–40 cm**									
CK	71.4a	24.2a	3.7a	0.09a	48.4a	19.1b	0.015a	–	47.9a
NPK	72.6a	23.6a	3.4a	0.10a	46.2ab	20.9b	0.017a	0.037a	45.6ab
2NPK	72.4a	23.8a	2.9a	0.12a	44.5ab	22.2ab	0.016a	0.017b	44.1ab
MNPK	71.8a	24.2a	2.7a	0.12a	41.8b	25.5a	0.018a	0.038a	41.5b

Each value is the mean of three replicates. Different letters within the same column indicate the least significant difference values at the 0.05 level (LSD0.05) for the different treatments at the different depths.

Macro, free-m, and free-SC are macroaggregates (> 250 µm diameter), microaggregates (53–250 µm diameter), and silt plus clay (< 53 µm diameter), respectively. cPOM, inter-m, and inter-SC are coarse particulate organic matter (POM), microaggregates in macroaggregates, and silt–clay in macroaggregates, respectively. fPOM, fiPOM, and intra-SC are fine POM, fine intra-POM, and intra-silt–clay in inter-m, respectively. Inter-m is the sum of fPOM, fiPOM, and intra-SC. fiPOM was not detected at 20–40 cm deep.

**Figure 1:**
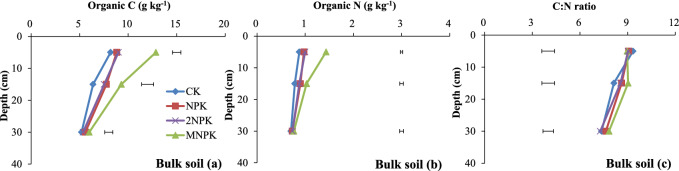
Organic C and N concentrations and C/N ratios in bulk soil and water-stable aggregate fractions to 40 cm deep in the control treatment (CK), chemical fertilizer treatments (NPK and 2NPK), and combined manure and chemical fertilizer treatment (MNPK). Each value is the mean of three replicates. The horizontal lines indicate the least significant difference values at the 0.05 level (LSD0.05) for the different treatments at the different depths. The abbreviations for aggregates of each size are shown in the footnote to Table 1.

The SOC and SON concentrations were significantly higher (P < 0.05) for the MNPK treatment than for the other three treatments at 0–10 and 10–20 cm deep (Fig. 1a, b). The SOC and SON concentrations at 10–20 cm deep and SON concentrations at 0–10 cm deep were significantly higher (P < 0.05) for the chemical fertilizer treatments than for the control (Fig. 1a, b). The C/N ratio in the bulk soil at 10–20 cm deep was significantly higher (P < 0.05) for the MNPK treatment than for the control (Fig. 1c).

**Figure 2:**
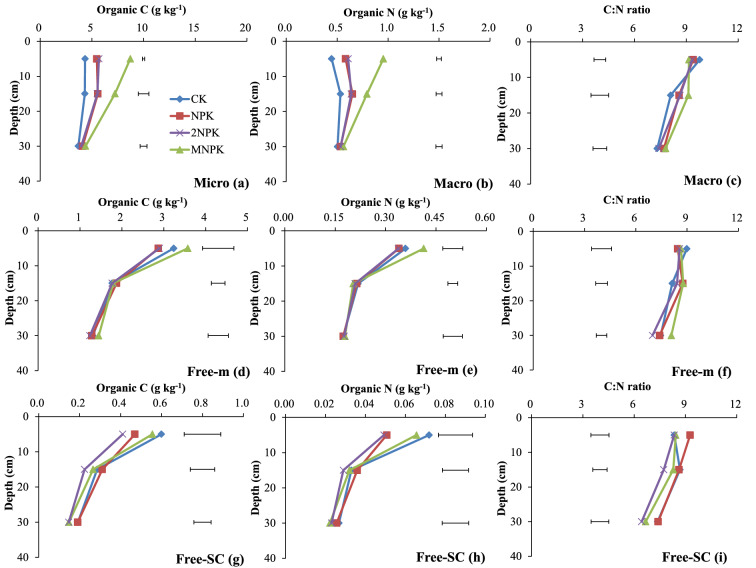
Organic C and N concentrations and C/N ratios of the water-stable aggregate fractions to 40 cm deep in the control treatment (CK), chemical fertilizer treatments (NPK and 2NPK), and combined manure and chemical fertilizer treatment (MNPK). Each value is the mean of three replicates. The horizontal lines indicate the least significant difference values at the 0.05 level (LSD0.05) for the different treatments at the different depths. The abbreviations for aggregates of each size are shown in the footnote to Table 1.

At 0–10 and 10–20 cm deep, the C and N concentrations in the macroaggregates were significantly higher (P < 0.05) for the fertilizer treatments than for the control. The C and N concentrations of the macroaggregates were significantly higher (P < 0.05) for the MNPK treatment than for the chemical fertilizer treatments (Fig. 2d, e). At 0–10 cm deep, the N concentration of free-m were significantly higher (P < 0.05) for the MNPK treatment than for the chemical fertilizer treatments (Fig. 2e). The C and N concentrations in free-SC were significantly higher (P < 0.05) for the control than for the chemical fertilizer treatments except for the C concentration in free-SC in the NPK treatment (Fig. 2g, h).

The C/N ratio of free-SC at 10–20 cm deep was significantly higher (P < 0.05) for the control than for the 2NPK treatment (Fig. 2i), and the C/N ratio of free-m at 20–40 cm deep was significantly higher (P < 0.05) for the MNPK treatment than for the other three treatments (Fig. 2f).

**Figure 3:**
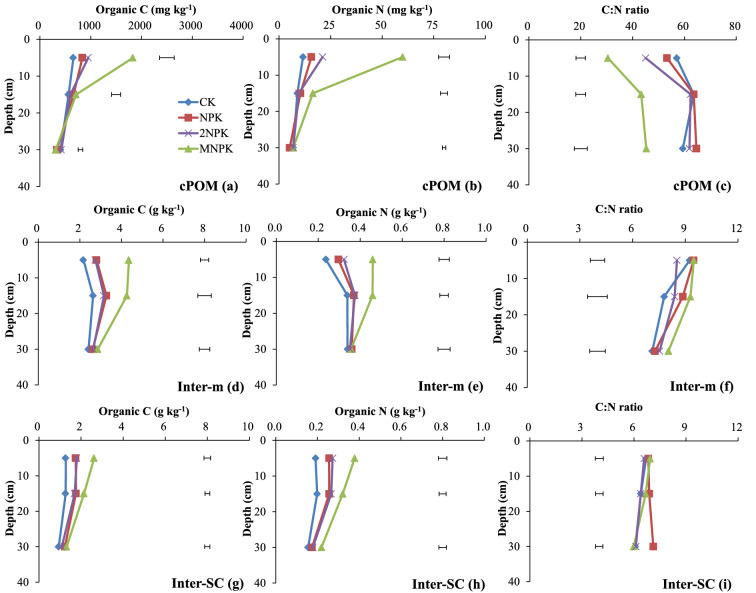
Organic C and N concentrations and C/N ratios of the macroaggregate fractions to 40 cm deep in the control treatment (CK), chemical fertilizer treatments (NPK and 2NPK), and combined manure and chemical fertilizer treatment (MNPK). Each value is the mean of three replicates. The horizontal lines indicate the least significant difference values at the 0.05 level (LSD0.05) for the different treatments at the different depths. The abbreviations for aggregates of each size are shown in the footnote to Table 1.

The C and N concentrations in cPOM, inter-m, and inter-SC at 0–10 cm deep and inter-m and inter-SC at 10–20 cm deep and the N concentrations in cPOM at 10–20 cm deep and inter-SC at 20–40 cm deep were significantly higher (P < 0.05) for the MNPK treatment than for the other three treatments (Fig. 3). The C and N concentrations in inter-m and inter-SC at 0–10 deep and inter-SC at 10–20 cm deep were significantly higher (P < 0.05) for the chemical fertilizer treatments than for the control.

The C/N ratios of cPOM at 0–10 cm deep were significantly lower (P < 0.05) for the fertilizer treatments than for the control (Fig. 3c). The C/N ratios of cPOM to 40 cm deep were significantly lower (P < 0.05) for the MNPK treatment than for the other treatments (Fig. 3c). In most cases at each depth, the C/N ratios of inter-m and inter-SC were higher for the MNPK treatment than for the other three treatments except for inter-SC at 20–40 cm deep. The C/N ratio of inter-m at 10–20 cm deep was significantly higher (P < 0.05) for the MNPK treatment than for the control (Fig. 3f, i). In the macroaggregate fractions, the C/N ratio was usually higher for the NPK fertilizer treatment than for the 2NPK fertilizer treatment, and the C/N ratios of cPOM and inter-m at 0–10 cm deep and of inter-SC at 10–20 and 20–40 cm deep were significantly different between the NPK and 2NPK treatments (P < 0.05) (Fig. 3c, f, i).

**Figure 4:**
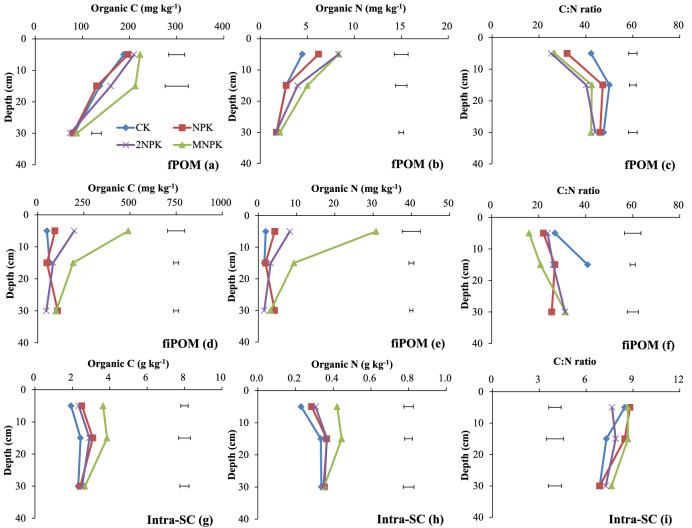
Organic C and N concentrations and C/N ratios in microaggregates in the macroaggregate fractions to 40 cm deep in the control treatment (CK), chemical fertilizer treatments (NPK and 2NPK), and combined manure and chemical fertilizer treatment (MNPK). Each value is the mean of three replicates. The horizontal lines indicate the least significant difference values at the 0.05 level (LSD0.05) for the different treatments at the different depths. Inter-m is the sum of fPOM, fiPOM, and intra-SC. fPOM was not detected at 20–40 cm deep. The abbreviations for aggregates of each size are shown in the footnote to Table 1.

The C and N concentrations in fiPOM and intra-SC at 0–10 cm deep and fiPOM and intra-SC at 10–20 cm deep and the C concentration in fPOM at 10–20 cm deep were significantly higher (P < 0.05) for the MNPK treatment than for the other three treatments (Fig. 4a, b, d, e, g, h). The C and N concentrations in intra-SC at 0–10 deep were significantly higher (P < 0.05) for the chemical fertilizer treatments than for the control (Fig. 4g). The C concentration in fiPOM to 20 cm deep (Fig. 4d) and the N concentration in fPOM at 0–10 cm deep and fiPOM at 10–20 cm deep (Fig. 4b, e) were significantly higher (P < 0.05) for the 2NPK treatment than for the NPK treatments.

The C/N ratios of fPOM and fiPOM at each depth (Fig. 4c, f) decreased as the fertilizer application rate increased. The C/N ratios of fPOM at 0–10 cm deep (Fig. 4c) and of fPOM and fiPOM at 10–20 cm deep (Fig. 4c, f) were significantly lower (P < 0.05) for the fertilizer treatments than for the control. The C/N ratios of fPOM at 0–10 and 10–20 cm deep were significantly lower (P < 0.05) for the 2NPK treatment than for the NPK treatment (Fig. 4c). The C/N ratio of intra-SC at 0–10 cm deep was significantly lower (P < 0.05) for the 2NPK treatment than for the other treatments. The C/N ratio of intra-SC at 10–20 cm deep was significantly higher (P < 0.05) for the MNPK treatment than for the control (Fig. 4i).

**Figure 5:**
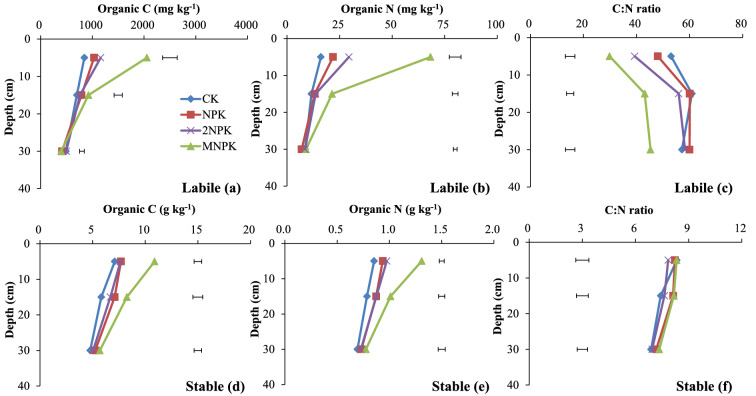
Organic C and N concentrations and C/N ratios in the labile and stable fractions to 40 cm deep in the control treatment (CK), chemical fertilizer treatments (NPK and 2NPK), and combined manure and chemical fertilizer treatment (MNPK). The C or N concentration of the labile fraction is the sum of the C or N concentrations of cPOM and fPOM. The C or N concentration of the stable fraction is the sum of the C or N concentration of fiPOM, free-m, free-SC, inter-SC, and intra-SC. Each value is the mean of three replicates. The horizontal lines indicate the least significant difference values at the 0.05 level (LSD0.05) for the different treatments at the different depths. The abbreviations for aggregates of each size are shown in the footnote to Table 1.

The C and N concentrations in the labile pool at 0–10 and 10–20 cm deep and in the stable pool at each depth were significantly higher (P < 0.05) for the MNPK treatment than for the control (Fig. 5a, b, d, e). The N concentrations in the labile pool (Fig. 5b) and the C and N concentrations in the stable pool at 0–10 and 10–20 cm deep were significantly higher (P < 0.05) for the MNPK treatment than for the chemical fertilizer treatments (Fig. 5d, e).

The C/N ratio of the labile pool at 0–10 and 10–20 cm deep except for the control and NPK treatments at 10–20 cm deep decreased significantly (P < 0.05) in the following order: control > NPK > 2NPK > MNPK (Fig. 5c). The C/N ratio of the stable pool at 10–20 cm deep was significantly higher (P < 0.05) for the MNPK treatment than for the control (Fig. 5f).

Analysis of variance indicated that soil depth significantly (P < 0.001) affected the masses (not including bulk soil), C concentrations, N concentrations, and C/N ratios of the bulk soil, labile pool, stable pool, and aggregates of different sizes (Table 2). The fertilization practice non-significantly affected the mass of the stable pool and the intra-SC, the C and N concentrations and C/N ratios of free-m, the N concentration of free-SC, and the C/N ratios of the microaggregates.

**Table 2:** Analysis of variance results for the effects of the fertilization practices, soil depth, and their interactions on the mass, C concentration, N concentration, and C/N ratio in bulk soil and aggregates of different sizes.

	Bulk soil	Labile pool	Stable pool	Water stable aggregates			Macroaggregates			Density fractionation		
				Macro	Free-m	Free-SC	cPOM	Inter-m	Inter-SC	fPOM	fiPOM	Intra-SC
**Mass**												
Fertilization (F)	–	***	n.s	***	**	**	***	n.s	***	***	***	n.s
Soil depth (D)	–	***	***	***	***	***	***	***	***	***	***	***
F × D	–	***	n.s	*	n.s	n.s	***	n.s	n.s	***	***	n.s
**C concentration**												
Fertilization (F)	***	***	***	***	n.s	*	***	***	***	**	***	***
Soil depth (D)	***	***	***	***	***	***	***	***	***	***	***	***
F × D	***	***	***	***	n.s	n.s	***	**	***	*	***	**
**N concentration**												
Fertilization (F)	***	***	***	***	n.s	n.s	***	***	***	***	***	***
Soil depth (D)	***	***	***	***	***	***	***	***	***	***	***	***
F × D	***	***	***	***	n.s	n.s	***	***	**	***	***	***
**C/N ratio**												
Fertilization (F)	n.s	***	*	n.s	n.s	**	***	*	***	***	***	*
Soil depth (D)	***	***	***	***	***	***	***	***	**	***	***	***
F × D	n.s	**	n.s	n.s	n.s	n.s	*	n.s	**	**	***	n.s

*Macro* macroaggregates, *Free-m* free macroaggregates, *Free-SC* free silt–clay particles, *cPOM* coarse particulate organic matter, *inter-m* microaggregates within macroaggregates, *Inter-SC* silt–clay particles in macroaggregates, *fPOM* fine particulate organic matter, *fiPOM* fine intra particulate organic matter, *Intra-SC* silt–clay in inter-m.

*Indicates that a difference was found by performing the ANOVA at P < 0.05, ** indicates that a difference was found by performing the ANOVA at P < 0.01, *** indicates that a difference was found by performing the ANOVA at P < 0.001, n.s. indicates that no significant difference was found by performing the ANOVA at P > 0.05.

Interactions between soil depth and fertilization practice significantly (P < 0.05 or P < 0.001) affected the masses of the labile pool, macroaggregates, and POM fractions, i.e., cPOM, fPOM, and fiPOM. Interactions between soil depth and fertilization practice non-significantly affected the C and N concentrations in free-m and free-SC and the N concentration of the bulk soil. Interactions between soil depth and fertilization practice significantly (P < 0.05, P < 0.01, or P < 0.001) affected the C/N ratios of the labile pool, POM fractions, and inter-SC.

**Figure 6:**
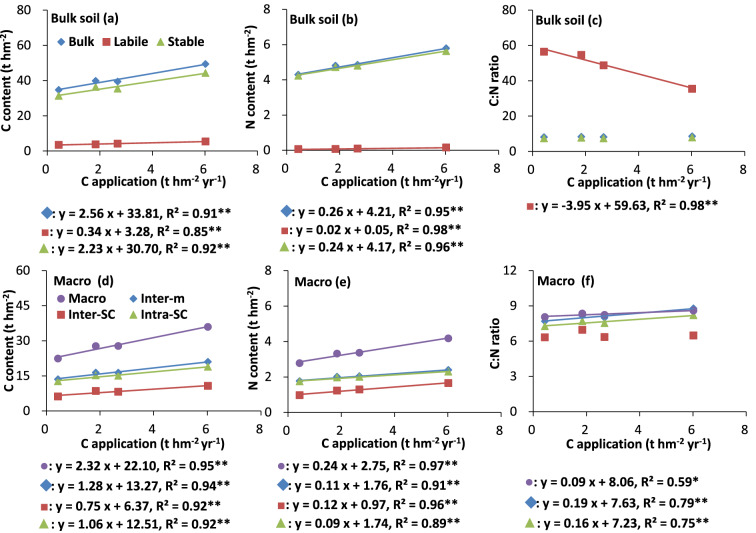
Relationships between carbon inputs and carbon stocks in different fractions above 40 cm soil depth in different treatments. Values are the mean of three replicates. Carbon input sequence on the x-axis is as follows: CK, NPK, 2NPK, and MNPK treatments. * and ** indicate significant difference at P < 0.05 and P < 0.01, respectively. R^2^ value was calculated using all the replicates of treatments. See footnote of Table 1 for abbreviations for aggregate fractions.

The C and N stocks in the bulk soil, labile pool, and stable pool significantly (P < 0.05) linearly increased as the annual C input rate increased (Fig. 6a, b). The C and N stocks in the stable pool contributed most of the C and N stocks in the bulk soil (Fig. 6a, b). The C and N stocks in the macroaggregates and the macroaggregate fractions significantly (P < 0.05) linearly increased as the annual C input rate increased, as shown for inter-m, inter-SC, and intra-SC in Fig. 6d, e.

The C/N ratios of the macroaggregates, inter-m, and intra-SC were significantly (P < 0.05) positively linearly related to the increase in the annual C input rate (Fig. 6f). The C/N ratios of the labile pool significantly (P < 0.05) linearly decreased as the annual C input rate increased (Fig. 6c).

## Discussion

### C and N concentrations of the bulk soil

Previous studies have shown that an increase in the C supply increases the C and N contents in bulk soil^4^, consistent with our results (Fig. 6a, b). Crop residues and roots were the main sources of C in the control and chemical fertilizer treatments. However, manure provides additional C and N and further enhances organic C and N accumulation when it is applied in combination with chemical fertilizer to the soil^4^. This explains why, in the present study, the SOC and SON concentrations at 0–10 and 10–20 cm deep were highest in the MNPK treatment and the lowest in the control (Fig. 1a, b). The crop biomass generally increases as the N fertilizer application rate increases until the optimal N application rate is reached^30^. In our study, however, the SOC and SON concentrations for the NPK and 2NPK treatments were generally similar (Fig. 1a, b). This may have been because: (1) the additional N added in the 2NPK treatment could not be immobilized in the short term because of a lack of available C; and (2) nitrate was leached during abundant rainfall after the applied urea had been nitrified. With increasing soil depth for each treatment, the lack of C input from crop residue resulted in significant decreases (P < 0.05) in SOC and SON concentrations in the subsurface soil compared with the surface soil (Fig. 1a, b; Table 2).

### C and N concentrations and masses in the aggregates

Macroaggregates are complex mixtures of the labile pools and part of the stable pools of SOM. Macroaggregate formation and microaggregate binding to macroaggregates are expected to increase the SOM content^31,32^. Crop residues and manure provide C for microorganisms and promote macroaggregate formation through mineral particles and small aggregates forming associations^18,33^. Consequently, the C and N concentrations and masses in the macroaggregates and macroaggregate fractions at 0–10 and 10–20 cm deep were significantly higher (P < 0.05) for the MNPK treatment than for the other treatments (Fig. 2a, b; Table 1).

Tilling the surface soil in agricultural land can break down macroaggregates into small particles and expose the SOM to microbial attack^9,10,34,35^. Thus, tilling explains the lower macroaggregate masses at 0–10 cm deep than at 10–20 and 20–40 cm deep for all four treatments (Table 1). In soil, SOC acts as binding agent for aggregate formation and stabilization, inter-m is the important fractions for the physical protection of SOC, and macroaggregates hold inter-m together with binding agents^18,36^. This explains why abundant exogenous C applied to surface soil increased the C and N concentrations in the macroaggregates and macroaggregate fractions at 0–10 cm deep relative to below 10 cm deep, particularly for the MNPK treatment (Fig. 2a, b).

The POM is an index for the labile C and N pools and is an important agent for binding macroaggregates^18,37^. Decomposition of cPOM via fPOM gives fiPOM, and the cPOM content reflects the quantity and quality of the original C added to soil^17,26^. Consequently, the greater C and N inputs in the MNPK treatment than in the other treatments resulted in significantly higher (P < 0.05) C and N concentrations in the cPOM, fiPOM, and labile pool to 20 cm deep in the MNPK treatment (Figs. 3a, b, 4d, e, 5a, b). Both fPOM and fiPOM in microaggregates within macroaggregates strongly contribute to increased SOC stocks^20,38^. The MNPK treatment showed obviously higher C and N concentrations and greater mass of fiPOM than fPOM (Fig. 4) at 0–10 cm deep, this was because: (1) fiPOM was denser than fPOM, and fiPOM associated with mineral particles more stably than fPOM^39^; (2) fPOM was more readily decomposed and exposed to microorganisms than fiPOM during soil tilling^32,40^ and (3) manure per se has plenty of passive organic matter.

The soil aggregate fractions except for the labile pool are stable and are responsible for storing and stabilizing C and N^17,34,37^. When sufficient exogenous C is supplied, microaggregates and mineral particles bind together to form macroaggregates, thereby decreasing the number of soil free particles (free-m and free-SC). This situation promotes the accumulation of SOM that resists microbial mineralization of C and N^10,41^. This explains why the C and N concentrations in the stable pool, inter-m, inter-SC and intra-SC at 0–10 and 10–20 cm deep significantly increased (P < 0.05) as the fertilizer application rate increased (Figs. 3d, e, g, h, 4g, h, 5d, e), and why those in free-m and free-SC at 0–10 cm deep were significantly lower (P < 0.05) in the fertilizer treatments than in the control (Fig. 2d, e, g, h).

Different from surface soil, in subsurface soil the C and N inputs are mainly from degraded or leached compounds of exogenous substrates and root residues^4^. In the present study, the mass, C concentration, and N concentration of each aggregate fraction differed significantly (P < 0.05) among soil depths and fertilization practices, and were affected by soil depths × fertilization practices interaction (Table 2; Figs. 1, 2, 3, 4, 5). This was attributable to differences in the C and N input rates, and the biological, physical, and chemical associations between degraded or leached small molecular compounds and soil particles*.*

The studied red soil had high C and N sequestration potential, as indicated by the significant (P < 0.05) linear increase in the C and N contents in various soil fractions to 40 cm deep as the C input rate increased (Fig. 6). King et al.^42^ found that the C and N sequestration potentials of soil aggregates are closely related to the aggregate mass, the C and N contents of the aggregates, and the amount and capacity of the SOM associated with the soil particles.

### C/N ratio

The C/N ratio in bulk soil is generally constant^25^. In the acidic soil studied here, N immobilization is not only regulated by the availability and amount of exogenous C, but also affected by the ferrous cycle^43^. Both of these reasons may explain the slight decreases in the C/N ratios in the bulk soil and all of the aggregate fractions except for POM as the soil depth increased (plot c, f, i in Figs. 2, 3, 4, 5).

The C/N ratio of POM reflects the original characteristics and decomposability of the exogenous C^17,26^. The decreasing C/N ratio from cPOM to fiPOM via fPOM (Figs. 3c, 4c, f) indicated that, among the POM fractions, fiPOM was composed of the most decomposable, oldest, and least energy-rich materials^26^. The C/N ratios of the POM fractions and labile pool were higher in the control and lower in the MNPK treatment (plot c in Figs. 3, 4, 5, 6; Fig. 4f), because the C/N ratio of manure is lower than that of maize residues^26^. The higher C/N ratios for cPOM and fPOM at 10–20 cm deep than at the other deeps may have been related to more organic C in POM being mineralized or more N being microbially immobilized at 0–10 cm deep than in deeper layers (Figs. 3c, 4c).

The C/N ratios in aggregate fractions in the stable pool stabilize the C/N ratio of the bulk soil^25^, as reflected by the similar C/N ratios between the aggregate fractions in the stable pool and the bulk soil (Figs. 1, 2, 3, 4, 5, 6). Even so, different fertilization practices affected the C/N ratios in aggregate fractions in the stable pool. For example, the C/N ratios were significantly higher (P < 0.05) for inter-SC and intra-SC at 0–10 cm deep (Figs. 3i, 4i) for the MNPK treatment than for the 2NPK treatment because sufficient C supply in the MNPK treatment promotes macroaggregates formation and SOC physical protection in macroaggregates^18^; the significantly higher (P < 0.05) C/N ratios of free-SC and inter-SC at 10–20 and 20–40 cm deep for the NPK treatment than for the 2NPK treatment was because an N shortage before N is supplied at an optimal level causes roots to grow into deeper soil layers^44^.

The inter-SC may have been the main soil particle type involved in N immobilization in macroaggregates. There are three main lines of evidence for this: (1) The degree of occlusion increased from free-SC to intra-SC via inter-SC, following aggregate hierarchy theory, but the C/N ratio of inter-SC was 1.37 (− 0.25 to 2.44) lower than the C/N ratios of free-SC and intra-SC (Figs. 2, 3, 4i); (2) The lowest amounts of sand and oxygen in intra-SC among the three silt–clay particles as the degree of occlusion increased^42^ meant that a large proportion of the C was protected in intra-SC at all soil depths in all of the treatments (Fig. 4g); (3) Sorption and desorption of soil mineral nutrients occur to a much smaller extent in coarse fractions (free-SC) than in fine fractions with higher specific surface areas^9,10^.

## Conclusions

Increasing the C input rate by applying fertilizers increased the C and N contents of the bulk soil and aggregates of different sizes, especially the C and N concentrations to 20 cm deep in the MNPK treatment. Microaggregates were the dominant particles containing C and N stocks, and the soil to 40 cm deep could strongly sequester C and N, because the C and N contents were positively related with C input rate. In addition, the C and N stocks in aggregates of different sizes were mainly in the surface soil, but application of fertilizer decreased the C/N ratios of the POM fractions. Inter-SC may have been the main particle type that immobilized N, as indicated by the low C/N ratio. Overall, microaggregates are the main particles of soil C and N storage, and increasing deep soil C input is beneficial to promote C and N sequestration in dryland red soil.

## Materials and methods

### Site description

**Figure 7:**
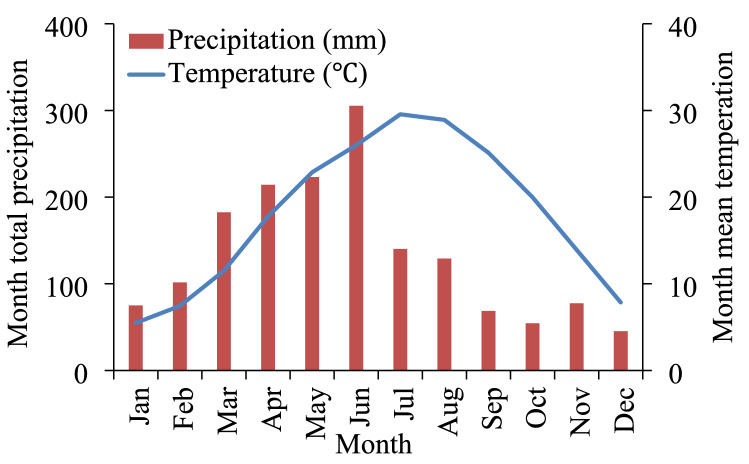
Monthly total precipitation and mean temperature from 1986 to 2015.

A long-term continuous maize cropping system was established at the study site (28°15′N, 116°20′E) in spring 1986. The study site was at the Red Soil Research Institute in Jiangxi Province, South China. The study site has a typical subtropical humid monsoon climate with a mean annual temperature of 18.1 °C and mean annual precipitation of 1620 mm (> 50% between March and July). The basic climate information is shown in Fig. 7. The soil at the study site is classed as Quaternary red soil in the Chinese soil classification system or as Ferralic Cambisol in the FAO soil classification system. The parent material of the soil is Quaternary red clay, and kaolinite is the dominant clay mineral. Fresh soil samples from 0–20 cm deep were collected before the experiment was started in 1986. Each sample was passed through a 2-mm sieve and then air-dried. The initial soil from 0–20 cm deep had a pH of 6.0, a SOC concentration of 9.4 g kg^−1^, and a total N concentration of 1.0 g kg^−1^, total P concentration of 1.4 g kg^−1^, total K concentration of 15.8 g kg^−1^, alkali-hydrolyzable N concentration of 60.3 mg kg^−1^, Olsen-P concentration of 12.9 mg kg^−1^, NH_4_Ac-K concentration of 102.0 mg kg^−1^, and comprised 21.2% sand, 27.6% clay, and 51.2% silt.

### Experimental design

The long-term continuous cropping maize experiment was performed using triplicate plots, each 22.2 m^2^ in area (5.05 m long × 4.0 m wide). An incompletely randomized block design was used. There were four treatments, (1) no fertilizer (control treatment), (2) chemical fertilizer treatment (NPK), (3) double rate of the chemical fertilizer treatment (2NPK), and (4) NPK and pig manure treatment (MNPK). The fertilizer application rates for the NPK treatment in each maize growing season were 60 kg N ha^−1^, 30 kg P_2_O_5_ ha^−1^, and 60 kg K_2_O ha^−1^. The fresh manure application rate for the MNPK treatment was 15,000 kg ha^−1^, which gave mean application rates of 1602 kg C ha^−1^ and 141 kg N ha^−1^. The chemical fertilizers that were used were urea, triple superphosphate or calcium superphosphate, and potassium chloride. The N fertilizer application during the crop growth period was split into 50% basal fertilizer and 50% topdressing fertilizer, with the latter applied when more than two-thirds of maize plants had six or seven leaves (about three weeks after seed emergence). Manure, P, and K were applied as basal fertilizers. Spring maize was sown at the beginning of April and harvested in the middle of July. Summer maize was then sown and harvested at the beginning of November. The maize cultivar Yedan 13 was grown with 50-cm spacing between rows and 30-cm spacing between plants within a row. After harvest, the maize residue was removed from plot. During maize growth, application of herbicide/pesticide and weed control were conducted manually as required. The maize was rain-fed because of the high precipitation in the subtropical region. Soil was ploughed by buffalo (to about 20-cm depth) in each season before the main crop was sown.

After the summer maize harvest in 2015, five soil core samples (40-cm depth) were collected from each plot using a tube sampler. Each core was divided into Sects. 0–10, 10–20, and 20–40 cm deep, then the same soil sections from the five core samples from each plot were mixed thoroughly to give composite samples for each depth. The bulk densities of the 0–10, 10–20, and 20–40 cm deep soil samples were 1.34, 1.40, and 1.43 g cm^−3^, respectively, for the control plots, 1.34, 1.48, and 1.48 g cm^−3^, respectively, for the NPK treatment plots, 1.40, 1.43, and 1.50 g cm^−3^, respectively, for the 2NPK treatment plots, and 1.35, 1.52, and 1.50 g cm^−3^, respectively, for the MNPK treatment plots. The masses of aggregates of different sizes and the C and N concentrations in the aggregates in soil from each depth from each plot were determined.

### Aggregate fractionation

Soil aggregates were separated using a size density fractionation method involving wet sieving and a heavy liquid [sodium polytungstate, Na_6_(H_2_W_12_O_40_)]. The method was based on methods previously described by Elliott^45^, Brown et al.^17^, and Gulde et al.^18^ reported. As described by Elliott^45^, water-stable aggregates were separated into three size classes, which were macroaggregates (> 250 µm diameter), free microaggregates (free-m) (250–53 µm diameter), and free silt–clay particles (free-SC) (< 53 µm diameter), using a wet sieving method. Briefly, 80.0 g of air-dried soil was placed in a 250 µm sieve and submerged in deionized water for 5 min at room temperature. The separation procedure involved 50 vertical movements in 2 min. Material that floated while the sample was submerged was removed using a net. The mean recovery efficiency of the wet sieving process was 98.8%, and the range was 97.7%–100.6%.

The microaggregates within macroaggregates were further isolated using a method described by Six et al.^32^. Briefly, ≤ 15.0 g of oven-dried macroaggregates was shaken for 15 min and then transferred to a 250 µm mesh screen with a transparent plastic wall. A total of 50 glass beads each 4 mm in diameter were placed on the screen, then the screen was shaken under a stream of water until the water ran clear. The < 250 µm soil slurry was passed through a 53 µm mesh screen using the wet sieving method to fractionate the soil into 53–250 µm and < 53 µm soil particles. The particles with diameter ranges > 250 µm, 53–250 µm, and < 53 µm were defined as coarse particulate organic matter plus sand (cPOM + sand), microaggregates within macroaggregates (inter-m), and silt–clay particles in macroaggregates (inter-SC), respectively. The mean recovery efficiency was 99.4%, and the range was 97.0%–101.7%.

The inter-m particles were divided into three types, fine POM (fPOM), fine intra-POM (fiPOM), and silt–clay particles in inter-m (intra-SC). A 3–5 g aliquot of inter-m was treated using a heavy liquid to remove fPOM, and the heavy residual fraction was mixed with 0.5% sodium hexametaphosphate at a soil:liquid w/v ratio of 1:3 and shaken for 12 h. The dispersed slurry was passed through a 53 µm sieve, and the > 53 µm and < 53 µm particles were defined as fiPOM + sand in inter-m and intra-SC particles. The mean recovery efficiency was 99.5%, and the range was 98.1%–100.0%.

Density flotation was performed using sodium polytungstate using a soil:liquid w/v ratio of 1:3^18^ to isolate fPOM from inter-m at a density of 1.85 g cm^−3^. Density flotation was then performed to separate soil cPOM + sand and fiPOM + sand in inter-m from the sand at a density of 2.3 g cm^−3^. Each fraction obtained using this method was placed on a 20-µm mesh screen and washed 7–10 times with deionized water using a vacuum filtration device.

After each fractionation step, the separated soil particles were oven-dried at 60 °C and then weighed using a balance with a precision of 0.01 or 0.0001 g.

### Sample analysis

Each oven-dried soil sample was passed through a 0.15 mm sieve, then the C and N concentrations in the soil were determined using a Macrocube CN analyzer (Elementar, Hanau, Germany). Alkali-hydrolyzable N was determined using the Mason jar diffusion method^46^. Total P and K were digested in a nickel crucible using sodium hydroxide at 750 °C. Olsen-P was extracted using 0.5 M NaHCO_3_, and exchangeable K was extracted using 1 M NH_4_OAc. The total P and Olsen-P concentrations in the extracts were determined using the molybdenum blue colorimetric method using a wavelength of 880 nm^47^. The total K and NH_4_Ac-K concentrations were determined by atomic absorption spectrophotometry^48^. Soil texture was determined with a laser particle size analyzer (LS13320, Beckman Coulter, Brea, CA, USA). The pH of each soil sample was determined after mixing the soil in water at a soil:liquid w/v ratio of 1:2.5. The bulk density of each soil sample was calculated from the dry weight and volume of the soil.

### Estimating C inputs

Inputs of C were mainly from exogenous C in fertilizer and C in crop residues, including roots and aboveground stubble (Rs). The amounts of C in the crop residues in the control and the chemical fertilizer treatments were calculated using the equation

$$ {\text{C}}\,{\text{input}}\,{\text{in}}\,{\text{crop}}\,{\text{residues}} = \left( {\left( {\left( {{\text{GY}}/{\text{HI}}} \right) \times \left( {{26}\% /{74}\% } \right) \times {93}\% } \right) + \left( {{\text{SY}} \times {\text{Rs}}} \right)} \right) \times \left( {{1}{-}{14}\% } \right) \times {44}.{4}\% $$

where GY, SY, HI, and Rs are the grain yield, straw yield, harvest index, and amount of aboveground stubble expressed as a percentage of the total aboveground material after the maize was harvested, respectively. The mean GYs for the control, NPK, 2NPK, and MNPK treatments were 1.64, 6.92, 10.06, and 10.57 t ha^−1^ a^−1^, respectively. The HI and Rs were 0.49 and 0.03, respectively. The ratio 26% / 74% was the ratio between the belowground and aboveground biomass. The value 93% was the amount of root biomass to 40 cm deep expressed as a percentage of the total root biomass. The value 14% was the mean moisture content of the crop samples. The value 44.4% was the mean C concentration of the maize.

The input of C in the MNPK treatment was defined as the sum of the C in manure and returned maize residues.

### Statistical analysis

Data are expressed below on an oven-dried basis. One-way analyses of variance were performed using SPSS 24.0 software. The mean values of the variables for the different treatments were compared using the least significant difference at the 5% level. Analyses of variance were performed using the fertilization practice and soil depth as fixed variables using the GLM procedure in SPSS 24.0 software. Changes in the C and N concentrations and C/N ratios in the 40 cm deep cores in response to C inputs were assessed using linear, quadratic, and logarithm functions using SPSS 24.0 software. The function that fitted the data best according to the coefficient R^2^ and the residual sum of squares was used to calculate a response curve.

### Statement

All the plant experiments were in compliance with relevant institutional, national, and international guidelines and legislation.
